# A Review of Biomonitoring for Atrazine and Atrazine Metabolites Using Blood, Urine, and Sweat-Based Assays

**DOI:** 10.3390/ijerph23030317

**Published:** 2026-03-04

**Authors:** Cecelia Zielke, Angela Garay, Ngaruiya Kariuki, Kaila Solo Wong, Shaan Gogna, Caitlyn Nguyen, Emily A. Lau, Joelle Ann Dualan, Katherine Callagy, Luke Charles Frozina, Risha S. Koparde, Ruier Fang, Sofia Jacik, Sukhad Mutatkar, Tyler Houston, Trang Thanh Ly, Vanessa Huynh, Victoria Fan

**Affiliations:** 1 Endocrinology Graduate Group, University of California-Berkeley, Berkeley, CA 94704, USA; algaray@berkeley.edu; 2 Department of Management, Policy, and Community Health, University of Texas Health Science Center at Houston (UTHealth Houston), Houston, TX 77030, USA; ngaruiya.kariuki@uth.tmc.edu; 3 Department of Chemical Biology, University of California-Berkeley, Berkeley, CA 94704, USA; kwong.88@berkeley.edu (K.S.W.); risha.koparde@berkeley.edu (R.S.K.); tyler.houston@berkeley.edu (T.H.); 4 Department of Environmental Science, Policy & Management, University of California-Berkeley, Berkeley, CA 94704, USA; sgogn003@berkeley.edu; 5 Department of Molecular and Cellular Biology, University of California-Berkeley, Berkeley, CA 94704, USA; caitng07@berkeley.edu (C.N.); emilylau@berkeley.edu (E.A.L.); joelledualan@berkeley.edu (J.A.D.); kcallagy@berkeley.edu (K.C.); lukefrozina@gmail.com (L.C.F.); ruier.fang@berkeley.edu (R.F.); sofiajacik@berkeley.edu (S.J.); sukhadmutatkar@berkeley.edu (S.M.); lytrang@berkeley.edu (T.T.L.); vhuynh05@berkeley.edu (V.H.); tfan17@berkeley.edu (V.F.)

**Keywords:** endocrine-disrupting chemicals, atrazine, toxicology, exposure assessment, microdialysis, urinalysis, blood test

## Abstract

**Highlights:**

**Public health relevance—How does this work relate to a public health issue?**

- Over ninety percent of the United States population is exposed to endocrine-disrupting chemicals, including atrazine, on a daily basis, resulting in adverse health outcomes and increased healthcare costs, including an estimated 90,000 deaths annually and a $39 billion per year loss in economic productivity.
- The prevalence of atrazine exposure is increasing, and less invasive methods for toxicological exposure assessment are needed. Reviewing concentrations of atrazine and atrazine metabolites in blood, urine, and sweat in occupational, environmental, and acute toxic exposure contexts will inform the development of sweat-based sensors for atrazine.

**Public health significance—Why is this work of significance to public health?**

- The average concentration of atrazine in urine for all studies included in the systematic review was 18.33 ng/mL. In blood, stratified by exposure context, the average blood concentration in acute poisoning patients was 261 ng/mL, where the average blood concentration in prenatal exposure was 31.68 ng/mL maximum. No studies examined the concentration of atrazine in sweat.
- Given that 6–16% of atrazine is absorbed dermally and metabolized by skin enzymes, sweat-based biomonitoring proves an attractive atrazine exposure assessment if additional research is conducted to refine assessments.

**Public health implications—What are the key implications or messages for practitioners, policy makers and/or researchers in public health?**

- Toxicological exposure assessments are currently dominated by routine blood panels and urine profiles, but less invasive, sweat-based testing will enable patients and clinicians to more frequently and easily monitor atrazine exposure and take steps to limit exposure to endocrine-disrupting chemicals, including atrazine.
- Additional randomized controlled trials are needed on atrazine concentrations in sweat to enable sweat-based sensing of atrazine and atrazine metabolites.

**Abstract:**

In current clinical medicine, urinary profiling and blood tests are the primary toxicological exposure assessments for endocrine-disrupting chemicals (EDCs), including atrazine. Recent research suggests that analog monitoring of EDC concentrations and metabolites in sweat may be a less invasive, yet equally reliable method for conducting toxicological exposure assessments. However, no systematic reviews have identified whether concentrations of atrazine in sweat serve as a valid biomarker of environmental exposure. Thus, we performed a systematic review of the peer-reviewed literature to assess (1) if there is a correlation between the concentration of atrazine present in blood and urine and evaluate the evidence for sweat-based biomonitoring and (2) whether atrazine concentrations in sweat are a reliable and valid measurement of atrazine exposure based on the current state of evidence in the peer-reviewed literature. Databases included PubMed, Embase, Google Scholar, Cochrane Central Register of Controlled Trials, ClinicalTrials.gov, and WHO Global Index Medicus. Stratified by exposure context, the average blood concentration of atrazine and atrazine metabolites in acute poisoning patients was 261 ng/mL, and the average blood concentration in prenatal exposure contexts was 31.68 ng/mL maximum in the included studies. While physicochemical properties of atrazine metabolites, particularly deisopropylatrazine (DIA), suggest potential suitability for sweat-based monitoring, empirical validation through controlled sweat collection studies is required before this approach can be recommended for clinical or occupational use. The results of the systematic review were heterogeneous, and a narrative review was conducted. To conclude, no studies have examined the concentration of atrazine in sweat and whether sweat can be used as a statistically valid toxicological assessment of atrazine exposure.

## 1. Introduction

### 1.1. Rationale

#### 1.1.1. Endocrine-Disrupting Chemicals

Endocrine-disrupting chemicals (EDCs) are “natural or man-made chemicals that may mimic or interfere with the body’s hormones, glands, and overall endocrine system function [1].” EDCs have numerous deleterious consequences on human health, including disruption of metabolism, reproduction, sexual development, neurodevelopment, and cognitive function [2]. EDCs have also been associated with the development of cancers of the reproductive system, with most evidence supporting an association between Perfluorooctane Sulfonate (PFOS) exposure and breast cancer incidence, and one study demonstrating a low association between EDCs and testicular cancer [3,4,5,6,7,8]. In addition, the impact of EDCs can also be multigenerational/epigenetic, as many EDCs can cross the placental barrier during pregnancy and interrupt normal fetal development [9,10].

According to the Endocrine Society, one of the world’s leading authorities on the endocrine system and metabolism, there are over 1000 EDCs [11]. The most well-studied endocrine disruptors in the current clinical literature, as defined by the National Institute of Environmental Health Sciences [1], include atrazine, bisphenol A (BPA), Dioxins, Perchlorate, Per- and polyfluoroalkyl substances (PFASs), Phytoestrogens, Polybrominated Diphenyl Ethers (PBDEs), Polychlorinated Biphenyls (PCBs), Triclosan, Phthalates, parabens, Dichlorodiphenyltrichloroethane (DDT), Chlorpyrifos, and Brominated Flame Retardants (BFRs) [1].

The prevalence of exposure to these EDCs is high in our modern environment. A recent study from the *Journal of Internal Medicine* estimated that over 90% of the United States population is exposed to EDCs daily, resulting in over 90,000 deaths annually and costing $39 billion per year in economic productivity [12]. In addition, low-level daily EDC exposure has caused an estimated $340 billion per year in combined healthcare costs and lost earnings [4].

Despite international reference ranges to limit exposure to specific EDCs, major U.S. medical authorities, including the Food and Drug Administration (FDA), have yet to formally establish safe clinical reference ranges for EDCs in blood, urine, sweat, and interstitial fluids for three reasons: (1) EDCs have low-dose effects, (2) EDCs have non-monotonic dose–response curves, and (3) individual variation in response to a single exposure level of an EDC is significant. To expand on these reasons, certain EDCs have low-dose effects or require very little exposure to the EDC to exert a large effect on a patient’s health. EDCs with low-dose effects include BPA, which has been correlated with negative health consequences at exposure rates of 0.2 ng/kg and consequently reported as a maximum safe exposure by the European Food Safety Authority [1,13,14]. In addition, EDCs can have non-monotonic dose–response curves, meaning that extremely low doses or extremely high doses of EDCs can negatively impact human health outcomes more than a moderate dose of an EDC [15]. For both of these reasons, safe EDC exposure limits or ranges can be hard to define in a traditional clinical range format. In addition, individual tolerance to EDCs has demonstrated high variation in the clinical literature, making it difficult to standardize safe EDC exposure reference ranges.

Because clinical reference ranges are difficult to establish and have not yet been executed on a national scale, monitoring individual patients’ EDC exposure rates and quantifying individual tolerance to EDCs is important. With monitoring, patients are more likely to reduce EDC exposure and increase EDC excretion when appropriate, both of which can be easily reduced via environmental/lifestyle changes. For instance, one study on a sample of 100 adolescent girls found that avoiding personal care products containing parabens for just 3 days reduces urinary paraben concentration by 43.9% [16]. Another study found that dietary avoidance of canned food, fast food, and take-out food can reduce urinary BPA levels by nearly half [17]. From a lifestyle perspective, although elimination of EDCs from the body occurs primarily through physiologic detoxification processes in the liver and kidneys, recent research has suggested that EDCs can also be eliminated from the body through sweat induction during exercise or exposure to high temperatures via saunas [18].

The recent literature suggests that concentrations of EDCs in sweat may be a better indicator of long-term toxin load than blood tests or urinalysis [18]. This is because many EDCs are lipophilic and often persist in sebaceous glands for longer periods of time than in blood or urine. To provide one example, lipophilic EDCs such as BPA are shown to accumulate in fat tissue and sebaceous glands, and the concentration of BPA secreted in sweat is consequently higher than the concentration identified in blood or urine in human subjects [18].

This systematic review aims to inform medical research professionals and medical practitioners as to whether the current literature supports the use of sweat as a less clinically invasive assessment of EDC exposure in clinical and at-home settings, with specific regard to the EDC atrazine. This review may also inform the development of technologies, such as sweat-based biosensors, to detect EDC exposure in human subjects.

#### 1.1.2. Atrazine

Atrazine (C_8_H_14_ClN_5_) is a nonvolatile, white powder with moderate solubility, low soil adsorption, and a half-life of 41–231 days [19,20]. The s-triazine ring of atrazine allows atrazine to bind to the quinone-binding protein on photosystem II, which makes it an effective pesticide for plant death via oxidative stress. In humans, atrazine is hypothesized to disrupt the hypothalamic control of the pituitary glands and ovaries by binding to estrogen and androgen receptors [21,22].

The physicochemical properties of atrazine make atrazine an attractive target for sweat-based biomonitoring. Atrazine has a lipophilicity of 2.7, as indicated by the octanol–water partition coefficient, and a molecular weight of 215.69 g/mol [19,20,22]. Compared to bisphenol A (BPA), an endocrine-disrupting chemical for which sweat-based toxicological exposure assessments have been heavily researched, atrazine has a higher water solubility and lipophilicity, making it less likely to accumulate in sebaceous glands and other fat deposits in the human body. To provide specific values, atrazine has a higher water solubility than BPA, with values of 33 mg/L and 120 mg/L, respectively. In addition, the lipophilicity of atrazine is often lower than that of BPA, which holds an octanol–water partition coefficient in the range of 2.7–3.4 [20,22]. However, the difference between the water solubility and lipophilicity is minor, considering that (1) other chemicals with similar water solubilities and lipophilicities to atrazine can accumulate in human fat depots, (2) atrazine has a similar dermal absorption rate to BPA and other EDC’s, and (3) atrazine is metabolized by skin microsomal enzymes into atrazine metabolites, which act as a simple target for sweat-based exposure assessments. Atrazine metabolites produced by skin microsomal enzymes include deisopropylatrazine (DIA) and diaminochlorotriazine (DACT) [22]. The metabolite DIA is a particularly attractive target for investigating sweat-based biomonitoring with a low water solubility and high lipophilicity of 3.197 mg/L and an octanol–water partition coefficient of 2.4, which theoretically supports DIA accumulation in sebaceous glands and potential excretion in sweat, although this has not been empirically validated in human studies [20,21].

With regard to exposure, atrazine is a broad-spectrum synthetic herbicide used in wheat, corn, and nut fields, with 64–80 million pounds used in the Midwest United States and 70,000–90,000 tons globally, although it has been banned in the European Union since 2003 [19,23]. Atrazine exposure occurs at 80 percent through ingestion, 0.3 to 5.1 percent dermally, and 0.01 mg/kg/day through inhalation [24]. Nonoccupational exposure in the U.S. is 1.8 to 6.1 μg/kg day, which correlates to less than 5 percent of the population; however, atrazine has been found in up to 41% of drinking-water wells in midwestern states, with an average concentration of 0.162 μg/L in Canada [25,26]. Incidence of exposure is emphasized in children of farmers due to hand–mouth contact, while maternal exposure is associated with low fetal birth weight and birth defects [27].

With regard to pharmacokinetics, atrazine is absorbed primarily through the gastrointestinal tract, while 6–16% of atrazine is absorbed dermally [27,28]. Atrazine is then metabolized primarily in the liver and eliminated through urine (1–2 days post-exposure) and feces (2–4 days post-exposure) [27,28]. In the liver, phase I dealkylation removes ethyl and isopropyl groups from atrazine using the cytochrome P450 enzymes, CYP1A2 and CYP3A4, forming the metabolites desisopropylatrazine (DIA), desethylazatrazine (DEA), and diaminochloroatrazine (DACT). Phase II glutathione (GSH) conjugation converts the byproducts into atrazine mercapturate and deethylatrazine mercapturate [29]. Significant portions of dermally absorbed atrazine are metabolized by skin microsomal enzymes, forming the metabolites deisopropylatrazine and 2-chloro-4,6-diamino-s-triazine [27,28]. Atrazine metabolites detected in urinary profiling are consistent with the atrazine metabolites that would theoretically be excreted in sweat based on pharmacokinetic principles, including diaminochlorotriazine (DACT), desethylatrazine (DEA), desisopropylatrazine (DIA), atrazine mercapturate (AM), and hydroxyatrazine and hydroxydesethylatrazine, although empirical validation through sweat analysis is required. Based on the pharmacokinetics of both dermal and urinary atrazine metabolism and past studies that have conducted urinary profiling for atrazine exposure, monitoring sweat for atrazine metabolites proves a theoretically feasible atrazine exposure assessment.

Regarding pharmacodynamics, atrazine acts as a low-binding-affinity agonist for G protein-coupled estrogen receptors (GPERs), which activate the extracellular signal-regulated kinase pathway in BG-1 cells and induce the expression of steroidogenic factor-1 (SF-1), leading to increased aromatase activity and the conversion of testosterone into estrogen [25,27]. The Environmental Protection Agency has set a benchmark dose of 2.42 mg/kg/day and a lifetime exposure of 0.1 mg/kg/day to be safe, although a maximum contaminant level of 3 ppb has been linked to the incidence of cancer [27]. Atrazine dealkylation occurs within 10.8 to 11.2 h of exposure, and metabolites are detected after 24–48 h in urine [26].

To provide an overview of the current research on toxicological assessments of atrazine in blood, sweat, interstitial fluid, and urine, urinalysis of atrazine mercapturate using liquid chromatographic–tandem mass spectrometry is the primary method used to detect atrazine exposure [22]. A National Health and Nutritional Examination Survey from 2001 to 2002 showed that the U.S. population had a detection level of below 0.3 μg/L [26]. Children were shown to have a detection level of 0.6 to 22 μg/g creatine [26]. Atrazine can also enter the bloodstream through adsorption—although at a low level—and has been found in mammary fat tissue and milk at 0.5–2.3 μg/g [27].

Atrazine has numerous deleterious health effects upon chronic exposure, including disrupted fat metabolism and distribution in numerous studies, with specific links to increased visceral fat accumulation [27]. Atrazine exposure has also been associated with higher diagnosis of certain cancers, specifically non-Hodgkin’s lymphoma, and formal diagnosis of metabolic syndrome [22,27].

### 1.2. Objectives

To date, no comprehensive, systematic analyses exist in the peer-reviewed literature that explore the reliability of using sweat concentrations of atrazine as a biomarker of toxicological exposure to atrazine. Through this systematic review of the existing clinical literature, we seek to identify (1) if there is a correlation between the concentration of atrazine present in blood and urine and evaluate the evidence for sweat-based biomonitoring and (2) whether atrazine concentrations in sweat are a reliable and valid measurement of atrazine exposure based on the current state of evidence in the peer-reviewed literature.

## 2. Methods

Methods followed the PRISMA 2020 Checklist for systematic review reporting which is accessible in Supplementary Materials.

### 2.1. Eligibility Criteria

The inclusion criteria included otherwise healthy, normal-weight adults (ages 18–64) of any biological sex with a BMI classification less than 30 kg/m^2^ and a sample size of at least fifteen persons to be included in the final systematic review. The population was not restricted to the United States of America, and papers from anywhere in the world were assessed. The exclusion criteria included populations with a formal diagnosis of cystic fibrosis, chronic kidney disease, and acute kidney injury and patients with any serious medical condition that may directly impair sweating ability or sweat rate, including lymphoma, hyperthyroidism, acromegaly, pheochromocytoma, and metabolic dysfunction-associated steatohepatitis (MASLD). In addition, we excluded studies in which patients reported the use of pharmaceutical drugs that impair or exacerbate perspiration, including the serotonin–norepinephrine reuptake inhibitors (SNRIs) duloxetine and venlafaxine.

For synthesis, studies were grouped based on which body fluids they assessed, with some studies assessing only blood, urine, interstitial fluid, or sweat, and other studies including multiple body fluids.

### 2.2. Information Sources

**Figure 1:**
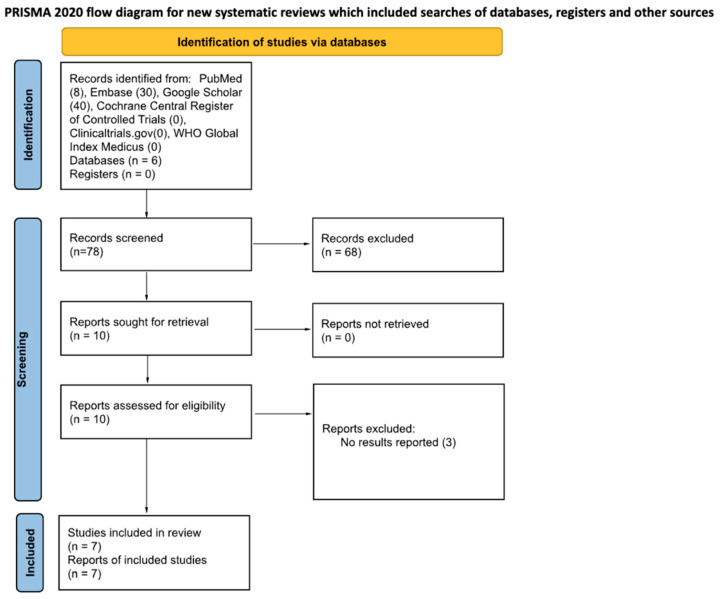
PRISMA 2020 flow diagram for the included studies.

The databases searched include PubMed, Embase, Google Scholar, Cochrane Central Register of Controlled Trials, ClinicalTrials.gov, and WHO Global Index Medicus. The number of search returns from each database is specified in Table 1. The reference lists of articles selected from the aforementioned databases were hand-searched for articles that met the inclusion criteria in several cases, and these studies are specified in the methods paragraphs where applicable (Figure 1).

### 2.3. Search Strategy

Searches were conducted using the advanced search function in each database. Six searches were performed using the keywords and the Boolean operators listed numerically below:

(1) (endocrine disruptors OR endocrine disrupting chemicals OR toxins OR toxic elements) AND (atrazine) AND (human OR humans OR human subjects);
(2) (endocrine disruptors OR endocrine disrupting chemicals OR toxins OR toxic elements) AND (atrazine) AND (human OR humans OR human subjects) AND (blood test OR blood OR hematologic test OR blood draw);
(3) (endocrine disruptors OR endocrine disrupting chemicals OR toxins OR toxic elements) AND (atrazine) AND (human OR humans OR human subjects) AND (urine OR urinalysis);
(4) (endocrine disruptors OR endocrine disrupting chemicals OR toxins OR toxic elements) AND (atrazine) AND (human OR humans OR human subjects) AND (sweat OR sweat collection);
(5) (endocrine disruptors OR endocrine disrupting chemicals OR toxins OR toxic elements) AND (atrazine) AND (human OR humans OR human subjects) AND (interstitial fluid OR microdialysis);
(6) (endocrine disruptors OR endocrine disrupting chemicals OR toxins OR toxic elements) AND (atrazine) AND (human OR humans OR human subjects) AND (blood test OR blood OR hematologic test OR blood draw) AND (urine OR urinalysis) AND (sweat OR sweat collection) AND (interstitial fluid OR microdialysis).

### 2.4. Selection Process

The methods used to decide whether a study met the inclusion criteria included reading through the full text of the study, paying specific attention to the sample selection section of the methods section of the study. Dual-independent screening was used to select studies included in the final systematic review. Two reviewers in total screened each study retrieved, working independently. No automation tools were used in the screening process. If there was disagreement between the two authors, results were discussed between the two authors. If there was no consensus after the initial discussion, a third author reviewed the study and determined whether the study met the inclusion criteria. Studies included in the final systematic review are recorded in Table 2.

### 2.5. Data Collection Process

Two reviewers collected data from each report, working independently. No processes to obtain or confirm data from study investigators were used. No automation tools were used in the process.

### 2.6. Data Items

Outcomes for which data were sought included (1) ranges of concentrations of atrazine in blood, urine, and/or sweat (2) comparison of the concentration of atrazine in sweat with the concentration of atrazine in blood and urine in human subjects, (3) whether atrazine concentrations in sweat were deemed a statistically valid measurement of atrazine exposure by each study’s authors, and 4) if atrazine concentration in sweat was deemed a reliable indicator of atrazine exposure, whether a safe exposure level for atrazine was defined in the study.

We did not reach out to the authors or co-authors of the studies to seek out missing participant and/or intervention characteristics or funding sources. No assumptions about missing or unclear information were made in the data extraction process for the included articles, and information that was missing and/or unclear in the study was noted as “not provided” in the tables and summary paragraphs for atrazine.

### 2.7. Study Risk of Bias Assessment

The methods used to assess risk of bias in the included studies were the Cochrane RoB 2 tool, or the updated Cochrane risk of bias tool designed to assess the potential for bias in randomized controlled trials (RCTs) included in systematic reviews, and the ROBINS-I tool, a tool used to assess the risk of bias in studies that evaluate the effects of interventions but do not use randomization to allocate participants. The Cochrane RoB 2 tool was used to assess risk of bias in randomized controlled trials, and the ROBINS-I tool was used to assess risk of bias in observational studies.

One reviewer completed a risk of bias assessment for each study. The risk of bias assessment for each study was then reviewed by a separate reviewer who had extracted data from the study. No automation tools were used in the process for risk of bias assessment.

### 2.8. Effect Measures

For each outcome, the average concentrations of metabolites in each body fluid for atrazine (blood, urine, sweat, ISF) for all studies found were included in units of either ng/mL or ng/g. For each study assessing multiple body fluids, the percentage of atrazine metabolites in sweat versus blood and/or the percentage of atrazine metabolites in sweat versus urine was calculated. For all included studies, the average percentage of atrazine metabolites in sweat versus blood and/or the average percentage of atrazine metabolites in sweat versus urine was calculated. For all calculations, risk ratios were also calculated.

Narrative synthesis was used to synthesize and present the results, as many of the studies were heterogeneous with regard to measurement instruments and measurement units and, therefore, presented different outcome measures.

### 2.9. Synthesis Methods

To decide which studies were eligible for each synthesis, both reviewers tabulated each study’s primary outcomes and matched the study into planned groups for each outcome specified in the Data Items Subsection of this review.

Concentrations of atrazines are presented in ng/mL or ng/g creatinine; the latter unit was used to account for urinary dilution. Atrazine concentrations not originally presented in ng/mL or ng/g in a study were converted to ng/mL or ng/g creatinine.

Calculation was performed for (1) the average concentrations of atrazine metabolites in each body fluid (blood, urine, sweat, ISF) for all studies found in the units of ng/mL or ng/g, (2) the ratio of atrazine metabolites in sweat versus blood for each study, (3) the ratio of atrazine metabolites in sweat versus urine for each study, (4) the pooled average ratio of atrazine metabolites in sweat versus blood for all studies included in the systematic review, and (5) the pooled average ratio of atrazine metabolites in sweat versus urine for all studies included in the systematic review.

In addition, the calculation of the odds ratio (OR) and risk ratio (RR) was performed for the following outcomes:

(1) When exposed to atrazine, whether the study concluded that sweat can be used to assess atrazine or atrazine metabolites load.
(2) When exposed to atrazine, whether the concentration of atrazine in sweat was greater than the concentration of atrazine or atrazine metabolites in blood.
(3) When exposed to atrazine, whether the concentration of atrazine in sweat was greater than the concentration of atrazine or atrazine metabolites in urine.

Finally, the standardized mean difference for concentrations of atrazine or atrazine metabolites was calculated in (1) sweat, (2) blood, and (3) urine for each study included in the final systematic review.

### 2.10. Meta-Analysis

A meta-analysis was not performed, as there was a significant amount of heterogeneity between studies.

### 2.11. Risk of Reporting Bias

Risk of bias arising from reporting bias was assessed in the reporting domain of the Cochrane RoB 2 tool and the ROBINS-I tool and reported in the tables denoting risk of bias for each EDC. The risk of reporting bias is recorded in Column 8 of Table 3 and Table 4 for non-randomized studies and randomized studies, respectively.

### 2.12. Certainty Assessment

The GRADE Scale was used to assess certainty in the body of evidence for the following outcomes: (1) there is a consistent difference in the concentrations of atrazine metabolites in sweat when compared to blood, (2) there is a consistent difference in the concentrations of atrazine metabolites in sweat when compared to urine, (3) there is a consistent difference in the concentrations of atrazine metabolites in sweat when compared to interstitial fluid, (4) sweat can be used as a body fluid when assessing the total load of atrazine in the body, (5) blood can be used as a body fluid when assessing the total load of atrazine in the body, and (6) urine can be used as a body fluid when assessing the total load of atrazine in the body. GRADE outcomes are presented in Table 5.

## 3. Results

The average concentration of atrazine in urine across all studies included in the systematic review was 18.33 ng/mL. Other major metabolites, such as atrazine deethylatrazine and atrazine mercapturic acid, were detected at an average of 12.8 ng/mL and 3.33 ng/mL, respectively. All studies reporting urinary concentrations of deethylatrazine and atrazine mercapturic acid included in the systematic review were included in the average calculations, except Perry et al. (2006), which received a critical risk of bias due specifically to performance bias, constituting a significant difference in exposure to atrazine between farmers assessed in the Perry et al. study [31]. Standard deviations could not be calculated since only ranges and means were reported for all studies included in the review, except Perry et al. [30] In this study, the pooled mean and standard deviation in urine for deethylatrazine, atrazine mercapturate, and triazines was 14.33 ± 12.0 ng/mL. Despite the reporting in Perry et al. [31] the lack of reported standard deviations compromises the variability in the concentrations of atrazine and atrazine metabolites and makes the predictability of similar measurement concentrations difficult to anticipate.

Stratified by exposure context, the average blood concentration in acute poisoning patients was 261 ng/mL, where the average blood concentration in prenatal exposure was 31.68 ng/mL maximum. The stratified arithmetic averages are limited in statistical power because only one study was included for each exposure context: one where 15 patients with acute poisoning were shown to have a maximum of 261 ng/mL detectable atrazine in human plasma [34] and another where 710 mothers had a maximum of 31.68 ng/mL atrazine in their blood [35]. To emphasize, pooling average blood concentrations from Yuan et al. [35] and Chevrier et al. [36] has limitations given that there is heterogeneity in both the study population and the exposure context: Yuan et al. [35] has a population with acute atrazine poisoning, and Chevrier et al. [36] has a population of pregnant women with atrazine exposure. Given the difference in exposure context, it may be more statistically accurate to consider the average blood concentrations of atrazine separately for the two studies rather than pooling the blood concentrations of atrazine. If considered separately rather than pooled as an average, the blood concentration found for acute poisoning patients was 261 ng/mL, and the average blood concentration for pregnant women with atrazine exposure was 31.68 ng/mL in our systematic review of the literature.

No studies measured the concentration of atrazine in sweat. Therefore, the ratio of atrazine metabolites in sweat versus blood could not be calculated. Similarly, no studies assessed the concentration of atrazine in multiple body fluids. The studies primarily focused on measuring atrazine in urine—the primary excretion route—and blood. Therefore, the ratio of atrazine metabolites in sweat as opposed to urine could not be calculated.

When assessing toxicity characteristics of atrazine in the included studies, it was found that atrazine’s lowest limit of quantification was 0.05 ng/mL, and its metabolite mercapturate could be detected at a low limit of 0.02 ng/mL. The non-monotonic dose–response curve for atrazine concentration in humans is not as thoroughly studied as it is in murine and amphibian models. Although the Environmental Protection Agency set a safe upper limit for atrazine in drinking water as 3 μg/L, its link to diseases like breast cancer and blood cancer is not strong enough to conclude atrazine’s true toxicity effect [37].

For ROBINS-I, 75% of the studies considered for atrazine were observational, given that the primary objective was to examine exposure in communities where there was a high risk of pesticide residuals leaking into ground and municipal water systems. Overall, 50% of the studies had a low overall risk of bias, while the remaining studies had a moderate risk of bias, with 67% of D2 and 83% of D5 marked as low risk. Notably, Perry et al. [30] was deemed to have a critical risk of bias since it failed to account for cofounding variables, which is reflected in its poor agreement between self-reporting and atrazine exposure.

Regarding Cochrane RoB 2 assessments, the two experimental studies for atrazine were both found to have a low risk of bias.

Finally, for GRADE outcomes, 60% of the studies that assessed urine as a viable body fluid to measure the total load of atrazine in the body had a high level of certainty, bolstered by their ability to represent a large effect in the population. Perry et al. [30] had a critical risk of bias, lowering its certainty to low overall, and Barr et al. [23] had a moderate risk of bias, resulting in a moderate certainty rating. All studies that verified blood as a body fluid to measure the amount of atrazine in the body had a high final level of certainty.

Due to the heterogeneity in exposure contexts in the studies included in the systematic review, a meta-analysis was not feasible. The implications of no meta-analysis are lower statistical power and precision compared to a systematic review in which a meta-analysis could be conducted. The inability to conduct a meta-analysis highlights the need for further studies on atrazine concentrations in blood, urine, and sweat in occupational, environmental, and acute poisoning exposure contexts.

## 4. Discussion

### 4.1. Assessing Safety of Observed Atrazine Concentrations in Blood and Urine

Given that the observed concentrations of atrazine and atrazine metabolites were higher than the estimated national average from NHANES data, further studies need to be conducted that stratify atrazine concentrations in urine and blood by exposure context, including environmental, occupational, and acute toxic exposures [20,28]. By stratifying atrazine concentrations by exposure context, more precise data for defining safe exposure limits for atrazine can be developed by healthcare governing bodies in the future. In this systematic review, the observed concentration of atrazine and atrazine metabolites in urine was much higher than the U.S. national average of 0.30 ng/mL [20,27,28]. The safe concentration of atrazine and atrazine metabolites in blood is not formally defined by a governing body, so a comparison to the average concentration of 261 ng/mL in the blood of acute poisoning patients and 31.68 ng/mL in the blood of prenatal exposure contexts cannot be made. While a 3.0 ng/mL safe exposure limit in drinking water has been set by the EPA, biomonitoring equivalent (BE) values have not been established to reliably convert concentrations of atrazine in drinking water to expected blood concentrations of atrazine due to heterogeneity in individual pharmacokinetics of atrazine [1,21,22].

Due to the lack of established limits for urinary concentrations and blood concentrations of atrazine and atrazine metabolites by major health governing bodies, it cannot be concluded whether the concentrations of atrazine in the populations of the studies included in this systematic review are safe. However, given that the concentrations of atrazine and atrazine metabolites detected in the studies are much higher than the U.S. national average for urinary excretion of atrazine and atrazine metabolites, the findings from this systematic review are likely not applicable to the general U.S. population. Rather, future studies need to be conducted in environmental and occupational exposure contexts to accurately assess and compare the concentrations of atrazine and atrazine metabolites in urine and blood in differential exposure contexts.

### 4.2. Assessing Feasibility of Sweat-Based Biomonitoring for Atrazine

Analog sweat monitoring of atrazine and atrazine metabolites is a limited area of study in clinical research to date, and sweat cannot be deemed a valid body fluid for measuring atrazine concentrations in the body at this time. While the primary route of exposure to atrazine in humans is absorption from the gastrointestinal tract into the bloodstream, and the primary method of atrazine excretion is likely through urine following dealkylation and glutathione conjugation in the liver, 6–16% of atrazine is absorbed dermally and can accumulate in sebaceous glands. In addition, dermal absorption is a primary route of occupational atrazine exposure, specifically for farm workers and herbicide applicators. Since atrazine is metabolized in the skin upon dermal exposure, sweat-based monitoring for atrazine metabolites has the potential to serve as a less invasive atrazine exposure assessment.

To conclude, more research needs to be conducted in clinical medicine on the rate and concentration of sweat-based excretion of atrazine metabolites, specifically controlled sweat collection studies in occupational cohorts with concurrent blood and urine sampling to establish sweat-to-blood ratios for atrazine metabolites. In addition, research should be conducted on whether sweat monitoring, either analog or real-time, may be a feasible method for performing toxicological exposure assessments for atrazine and EDCs generally, as there is a strong causal association between EDC exposure and various health conditions, as further detailed in Table 6 [38,39,40,41,42,43,44,45,46,47,48,49,50,51,52,53]. This manuscript represents one EDC (atrazine) from a larger registered review of all EDCs listed in Table 6, published separately due to data heterogeneity precluding combined analysis.

## 5. Conclusions

Following a systematic review of the literature on concentrations of atrazine and atrazine metabolites in urine, blood, and sweat, the concentrations of atrazine and atrazine metabolites in blood and urine varied by exposure context, and no studies measured atrazine and atrazine metabolites in sweat. When pooled, the average concentration of atrazine in urine across all studies included in the systematic review was 18.33 ng/mL. Other major metabolites, such as atrazine deethylatrazine and atrazine mercapturic acid, were detected in urine at an average of 12.8 ng/mL and 3.33 ng/mL, respectively. In blood, stratified by exposure context, the average blood concentration in acute poisoning patients was 261 ng/mL, whereas the average blood concentration in prenatal exposure was 31.68 ng/mL maximum. As previously emphasized, no studies in the current literature measured atrazine concentrations or atrazine metabolite concentrations in sweat. Ultimately, we highlight the evidence gap for monitoring atrazine exposure via sweat analysis. Despite a higher water solubility and lower lipophilicity than some of the most common EDCs, such as BPA, atrazine is an attractive EDC for sweat-based biomonitoring because atrazine has a similar dermal absorption rate to BPA and other EDCs. In addition, atrazine is metabolized by skin microsomal enzymes into atrazine metabolites, including deisopropylatrazine (DIA) and diaminochlorotriazine (DACT), which act as a simple target for sweat-based exposure assessments. The metabolite DIA is a particularly attractive target for investigating sweat-based biomonitoring, with a low water solubility and high lipophilicity of 3.197 mg/L and an octanol–water partition coefficient of 2.4, which may enable accumulation in sebaceous glands pending empirical validation [22]. We conclude that future epidemiological studies should be conducted to identify how concentrations of atrazine and atrazine metabolites vary in sweat in different exposure contexts, including environmental and occupational.

## 6. Other Information

Registration and Protocol: Comparison of atrazine concentrations in blood, sweat, urine, and interstitial fluid in human subjects: A systematic review and meta-analysis was initially registered as part of the systematic review Comparison of endocrine-disrupting chemical concentrations in blood, sweat, urine, and interstitial fluid in human subjects: A systematic review and meta-analysis in Prospero. The authors decided to publish an individual systematic review paper for each endocrine-disrupting chemical (EDC) included in the proposed review separately. This allowed for a meta-analysis to be performed on EDCs for which there was homogeneous data, and no meta-analysis was conducted for each EDC for which the systematic review searches returned heterogeneous data.

## Figures and Tables

**Table 1:** Search returns for atrazine.

Database	Number of Search Returns
PubMed	8
EmBase	30
Google Scholar	33,681
Cochrane Central Register of Controlled Trials	0
ClinicalTrial.gov	0
WHO Global Index Medicus	0

*The number of search returns for atrazine from each included database after applying the inclusion criteria. The twenty most recent records from each database were screened for inclusion in this systematic review.*

**Table 2:** Study characteristics of the included studies on atrazine.

Article Citation	Population Size and Exposure Context	Method of Detection (MD), Method Detection Limits (MDLs), and Limits of Quantification (LOQ)	Mean Concentration of Atrazine in Body Fluids (ng/mL)
Perry MJ, Christiani DC, Mathew J, Degenhardt D, Tortorelli J, Strauss J, et al. Urinalysis of atrazine exposure in farm pesticide applicators. *Toxicol Ind Health*. 2000 Aug 1;16(7–8):285–90 [30].	256	MD: GC-MS MDL: 1.0 ng/mL LOQ: not reported	14.2 ±13.5 ng/mL (deethylatrazine) 6.4 ± 7.5 ng/mL (atrazine mercapturate) 22.4 ± 13.9 ng/mL (triazines) in urine
Perry MJ, Marbella A, Layde PM. Nonpersistent pesticide exposure self-report versus biomonitoring in farm pesticide applicators. *Ann Epidemiol*. 2006 Sep;16(9):701–7 [31].	256	MD: GC-MS MDL: 1.0 ng/mL LOQ: not reported	8.03 ng/mL (deethylatrazine) in urine
Barr DB, Panuwet P, Nguyen JV, Udunka S, Needham LL. Assessing Exposure to Atrazine and Its Metabolites Using Biomonitoring. *Environ Health Perspect*. 2007 Oct;115(10):1474–8 [32].	24	MD: SPE-HPLC-MS/MS with isotope-dilution quantification MDL: 0.1–1.0 ng/mL LOQ: not reported	0.08 ng/mL (ATZ mercapturic acid) in urine
Cragin LA, Kesner JS, Bachand AM, Barr DB, Meadows JW, Krieg EF, et al. Menstrual cycle characteristics and reproductive hormone levels in women exposed to atrazine in drinking water. *Environmental Research*. 2011;111(8):1293 [33].	102	MD: HPLC-MS/MS MDL: 0.5 ng/mL (Atrazine, atrazine mercapturate, and desethylatrazine mercapturate), 1.0 ng/mL (desisopropylatrazine, desethylatrazine, and diaminochlorotriazine) LOQ: not reported	10.15 ng/mL (desethylatrazine mercapturate)
Wei H, Zhang X, Yang X, Yu Q, Deng S, Guan Q, et al. Prenatal exposure to pesticides and domain-specific neurodevelopment at age 12 and 18 months in Nanjing, China. *Environ Int*. 2023 Mar;173:107814 [34].	710 mother-child pairs.	MD: GC-MS/MS MDL: not reported LOQ: not reported	31.68 ng/mL (atrazine) in blood
Yuan G, Zhang R, Chen X, Wang B, Guo R. A simple and economical method of gas chromatography-mass spectrometry to determine the presence of 6 pesticides in human plasma and its clinical application in patients with acute poisoning. *Biosci Trends*. 2018;12(2):201–7 [35].	15	MD: GC-MS MDL: not reported LOQ: 0.05 µg/mL	261 ng/mL (atrazine) in plasma
Chevrier C, Limon G, Monfort C, Rouget F, Garlantézec R, Petit C, et al. Urinary biomarkers of prenatal atrazine exposure and adverse birth outcomes in the PELAGIE birth cohort. *Environ Health Perspect*. 2011 Jul;119(7):1034–41 [36].	256	MD: LC-MS/MS MDL: 0.015 ng/mL (atrazine), 0.006 ng/mL (atrazine mercapturate), 0.51 ng/mL (triazines and herbicides) LOQ: 0.05 ng/mL (atrazine), 0.02 ng/mL (atrazine mercapturate), 0.001–1.7 ng/mL (triazines and herbicides)	6.4 ng/mL (atrazine mercapturate) in urine 22.4 ng/mL (atrazine) in urine

**Table 3:** ROBINS-I risk of vias assessment results for atrazine.

Article Title	Domains of Bias							Overall Risk of Bias [Low, Moderate, Serious, or Critical]
	Confounding	Classification of IVs	Selection of Participants into the Study (or the Analysis)	Deviations from Intended Interventions	Missing Data	Measurement of the Outcome	Selection of Reported Results	
Perry MJ, Christiani DC, Mathew J, Degenhardt D, Tortorelli J, Strauss J, et al. Urinalysis of atrazine exposure in farm pesticide applicators. *Toxicol Ind Health*. 2000 Aug 1;16(7–8):285–90 [30].	LOW	LOW	LOW	LOW	LOW	MODERATE	LOW	MODERATE
Perry MJ, Marbella A, Layde PM. Nonpersistent pesticide exposure self-report versus biomonitoring in farm pesticide applicators. *Ann Epidemiol*. 2006 Sep;16(9):701–7 [31].	CRITICAL	MODERATE	LOW	LOW	LOW	LOW	LOW	CRITICAL
Barr DB, Panuwet P, Nguyen JV, Udunka S, Needham LL. Assessing Exposure to Atrazine and Its Metabolites Using Biomonitoring. *Environ Health Perspect*. 2007 Oct;115(10):1474–8 [32].	LOW	MODERATE	LOW	LOW	LOW	LOW	LOW	MODERATE
Cragin LA, Kesner JS, Bachand AM, Barr DB, Meadows JW, Krieg EF, et al. Menstrual cycle characteristics and reproductive hormone levels in women exposed to atrazine in drinking water. *Environmental Research*. 2011;111(8):1293 [33].	LOW	LOW	LOW	LOW	LOW	LOW	LOW	LOW
Wei H, Zhang X, Yang X, Yu Q, Deng S, Guan Q, et al. Prenatal exposure to pesticides and domain-specific neurodevelopment at age 12 and 18 months in Nanjing, China. *Environ Int*. 2023 Mar;173:107814 [34].	LOW	LOW	LOW	LOW	LOW	LOW	LOW	LOW
Chevrier C, Limon G, Monfort C, Rouget F, Garlantézec R, Petit C, et al. Urinary biomarkers of prenatal atrazine exposure and adverse birth outcomes in the PELAGIE birth cohort. *Environ Health Perspect*. 2011 Jul;119(7):1034–41 [36].	LOW	LOW	LOW	LOW	LOW	LOW	LOW	LOW

*Assessment of bias of non-randomized studies using the ROBINS-I tool for atrazine.*

**Table 4:** Cochrane RoB 2 risk of bias assessments for atrazine.

Article Title	Domains of Bias					
	Randomization Process	Deviations from Intended Interventions	Missing Outcome Data	Measurement of Outcomes	Selection of the Reported Result	Overall Risk of Bias [Low, Moderate, Serious, or Critical]
Yuan G, Zhang R, Chen X, Wang B, Guo R. A simple and economical method of gas chromatography-mass spectrometry to determine the presence of 6 pesticides in human plasma and its clinical application in patients with acute poisoning. *Biosci Trends*. 2018;12(2):201–7. [35]	LOW	LOW	LOW	LOW	LOW	LOW

*Assessment of bias in randomized controlled trials using the Cochrane RoB 2 tool for atrazine.*

**Table 5:** GRADE outcomes for atrazine.

Article Title	Domains of Bias				
	Outcome	Initial Certainty Level (RCT or Observational Study)	Lowering Domains Applied	Raising Domains Applied	Final Level of Certainty Rating
Perry MJ, Christiani DC, Mathew J, Degenhardt D, Tortorelli J, Strauss J, et al. Urinalysis of atrazine exposure in farm pesticide applicators. *Toxicol Ind Health*. 2000 Aug 1;16(7–8):285–90 [30].	Urine can be used as a body fluid when assessing total load of atrazine in the body.	LOW		LARGE EFFECT MODERATE ROB	HIGH
Perry MJ, Marbella A, Layde PM. Nonpersistent pesticide exposure self-report versus biomonitoring in farm pesticide applicators. *Ann Epidemiol*. 2006 Sep;16(9):701–7 [31].	Urine can be used as a body fluid when assessing total load of atrazine in the body.	LOW	CRITICAL ROB	LARGE EFFECT	LOW
Barr DB, Panuwet P, Nguyen JV, Udunka S, Needham LL. Assessing Exposure to Atrazine and Its Metabolites Using Biomonitoring. *Environ Health Perspect*. 2007 Oct;115(10):1474–8 [32].	Urine can be used as a body fluid when assessing total load of atrazine in the body.	LOW		MODERATE ROB	MODERATE
Cragin LA, Kesner JS, Bachand AM, Barr DB, Meadows JW, Krieg EF, et al. Menstrual cycle characteristics and reproductive hormone levels in women exposed to atrazine in drinking water. *Environmental Research*. 2011;111(8):1293 [33].	Urine can be used as a body fluid when assessing total load of atrazine in the body.	LOW		LOW ROB LARGE EFFECT	HIGH
Wei H, Zhang X, Yang X, Yu Q, Deng S, Guan Q, et al. Prenatal exposure to pesticides and domain-specific neurodevelopment at age 12 and 18 months in Nanjing, China. *Environ Int*. 2023 Mar;173:107814 [34].	Blood can be used as a body fluid when assessing total load of atrazine in the body.	LOW		LOW ROB LARGE EFFECT	HIGH
Chevrier C, Limon G, Monfort C, Rouget F, Garlantézec R, Petit C, et al. Urinary biomarkers of prenatal atrazine exposure and adverse birth outcomes in the PELAGIE birth cohort. *Environ Health Perspect*. 2011 Jul;119(7):1034–41 [36].	Urine can be used as a body fluid when assessing total load of atrazine in the body.	LOW		LOW ROB	HIGH
Yuan G, Zhang R, Chen X, Wang B, Guo R. A simple and economical method of gas chromatography-mass spectrometry to determine the presence of 6 pesticides in human plasma and its clinical application in patients with acute poisoning. *Biosci Trends*. 2018;12(2):201–7 [35].	Blood can be used as a body fluid when assessing total load of atrazine in the body.	HIGH		LOW ROB	HIGH

*Final GRADE outcomes for atrazine with indication of the initial certainty level, lowering domains, and raising domains applied to each study.*

**Table 6:** Examples of endocrine disruptors and strong causal links to specified health conditions.

Endocrine-Disrupting Chemical	Examples of Contamination Sources	Associated Diseases or Disorders
Bisphenol A (BPA)	Polycarbonate plastic: food containers, crockery, eyewear, cell phone and laptop screens Food can linings Building material: water supply pipes, grout, sealants	Type 2 diabetes, precocious puberty, decreased semen quality, hay fever, breast cancer, colorectal cancer [38,39,40,41]
Diethylstilbestrol (DES)	Livestock feed yielding food chain contamination like DES residue in beef	Clear cell vaginal carcinoma [42]
Dioxins (TCDD or 2,3,7,8-tetrachlorodibenzo-para-dioxin)	Contaminated animal feed yielding contaminated meat and dairy products Unregulated waste incineration	Female reproductive disorders (premature ovarian failure), impaired neurodevelopment, carcinogen (soft tissue sarcoma, non-Hodgkin’s lymphoma, lung cancer) [43,44,45,46]
Organophosphate pesticides	Run-off from agricultural fields into surface and groundwater	Cognitive deficits, neurodevelopmental delay, IQ loss [47,48,49]
Perfluoroalkyl and polyfluoroalkyl substances (PFASs)	Non-stick cookware, stain-resistant fabric, food packaging Soil and water contamination from manufacturing facilities and hazardous waste sites	Thyroid disorders: hypothyroidism, hyperthyroidism, papillary thyroid cancer [50,51]
Phthalates	Cosmetics: nail polish, shampoo, hair spray Construction material: vinyl flooring, wire insulation	Male reproductive disorders: decreased sperm count, hypospadias [52,53]

*Endocrine-disrupting chemicals (EDCs) are ubiquitous in the modern environment, making human exposure largely unavoidable. EDCs are globally found in the public water supply, food packaging, consumer goods (like plastic containers and non-stick pans), and soil.*

## Data Availability

We do not have any code or other materials that assisted in the authorship of this systematic review, other than those that are included in this systematic review and the reference list.

## Supplementary Materials

The following supporting information can be downloaded at: https://www.mdpi.com/article/10.3390/ijerph23030317/s1, Table S1: PRISMA 2020 Checklist [54].
